# Extracts from *Allium pseudojaponicum Makino* Target STAT3 Signaling Pathway to Overcome Cisplatin Resistance in Lung Cancer

**DOI:** 10.3390/md23040167

**Published:** 2025-04-14

**Authors:** Soo-Bin Nam, Jung Hoon Choi, Ga-Eun Lee, Jin Young Kim, Mee-Hyun Lee, Gabsik Yang, Yong-Yeon Cho, Hye Gwang Jeong, Geul Bang, Cheol-Jung Lee

**Affiliations:** 1 Biopharmaceutical Research Center, Korea Basic Science Institute (KBSI), Cheongju 28119, Republic of Korea; nsb0607@kbsi.re.kr (S.-B.N.); gelee3131@kbsi.re.kr (G.-E.L.); 2 College of Pharmacy, The Catholic University of Korea, Bucheon 14662, Republic of Korea; yongyeon@catholic.ac.kr; 3 Digital Omics Research Center, Korea Basic Science Institute (KBSI), Cheongju 28119, Republic of Korea; jhchoi19@kbsi.re.kr (J.H.C.); jinyoung@kbsi.re.kr (J.Y.K.); 4 College of Pharmacy, Chungnam National University, Daejeon 34134, Republic of Korea; hgjeong@cnu.ac.kr; 5 College of Korean Medicine, Dongshin University, Naju 58245, Republic of Korea; mhlee@dsu.ac.kr; 6 Department of Korean Medicine, College of Korean Medicine, Woosuk University, Jeonju 55338, Republic of Korea; yanggs@woosuk.ac.kr; 7 Department of Bio-Analytical Science, University of Science and Technology (UST), Daejeon 34113, Republic of Korea

**Keywords:** halophyte, *Allium pseudojaponicum Makino*, cisplatin resistance, non-small-cell lung cancer, STAT3 signaling pathway

## Abstract

Lung cancer, particularly non-small-cell lung cancer (NSCLC), remains a leading cause of cancer-related mortality, with cisplatin-based chemotherapy being a standard treatment. However, the development of chemoresistance significantly limits its efficacy, necessitating alternative therapeutic approaches. Here, we demonstrate the anticancer effects of the extracts of *Allium pseudojaponicum Makino* (APE), a salt-tolerant plant, in cisplatin-resistant NSCLC. Metabolite profiling using UPLC-Q-TOF-MS^E^ identified 13 major compounds, predominantly alkaloids (71.65%) and flavonoids (8.81%), with key bioactive constituents such as lycorine (29.81%), tazettine (17.22%), and tricetin (8.19%). APE significantly inhibited cell viability in A549 and H460 cells, reducing viability to 38.6% (A549-Ctr), 37.2% (A549-CR), 28.4% (H460-Ctr), and 30.4% (H460-CR) at 40 µg/mL after 48 h. APE also suppressed colony formation by over 90% in both 2D and soft agar assays, while showing no cytotoxicity in normal human keratinocytes up to 80 µg/mL. Flow cytometry analysis revealed APE-induced G1 phase arrest, with the G1 population increasing from 50.4% to 56.6% (A549-Ctr) and 47.5% to 58.4% (A549-CR), accompanied by reduced S phase populations. This effect was associated with the downregulation of G1/S transition regulators, including cyclin D1, CDK4, cyclin E, and CDK2. Furthermore, proteomic analysis identified STAT3 signaling as a major target of APE; APE decreased phosphorylated STAT3 and c-Myc expression, and STAT3 knockdown phenocopied the effects of APE. These findings highlight the potential of APE as a natural product-based therapeutic strategy for overcoming cisplatin resistance in NSCLC.

## 1. Introduction

Halophytes, a diverse group of salt-tolerant plants, have attracted considerable attention in recent years due to their potential applications in phytomedicine [1]. These plants thrive in high-salinity environments, such as coastal regions, salt marshes, and saline deserts, where most conventional plants cannot survive [2]. Their unique biochemical and physiological adaptations to extreme conditions result in a rich repository of bioactive compounds, positioning them as promising candidates for pharmaceutical and therapeutic research [3]. Among these, *Allium pseudojaponicum Makino*, a rare perennial plant belonging to the *Amaryllidaceae* family, is native to East Asia, specifically Japan and Korea [4]. This plant is found in coastal areas, highlighting its ecological significance as a halophyte. While previous studies on *A. pseudojaponicum* have largely focused on its germination characteristics, environmental adaptability, and potential anti-aging effects [4], its therapeutic potential in cancer treatment remains unexplored. Given the increasing interest in natural products for cancer therapy, this study aims to investigate the potential anticancer effects of *A. pseudojaponicum* extract (APE) in human lung cancer models, providing novel insights into its pharmacological applications and relevance to marine life science.

Lung cancer remains one of the most significant contributors to cancer-related mortality worldwide, with non-small-cell lung cancer (NSCLC) accounting for approximately 85% of all diagnosed cases [5]. Despite advancements in surgical techniques, radiation therapy, and systemic chemotherapy, the prognosis for lung cancer patients remains poor, particularly for those with advanced-stage disease [6]. A critical challenge in lung cancer management is the emergence of resistance to chemotherapeutic agents, such as cisplatin, which severely limits treatment efficacy [7]. Cisplatin, a platinum-based chemotherapeutic agent, has been a cornerstone in the treatment of various malignancies, including NSCLC. Its cytotoxic effect is primarily mediated through the formation of DNA cross-links, which inhibit DNA replication and transcription, ultimately inducing apoptosis [8]. While initially effective, the development of cisplatin resistance is a common phenomenon in lung cancer, driven by complex mechanisms such as enhanced DNA repair capacity, alterations in drug uptake and efflux, and activation of survival signaling pathways [9]. This resistance poses a significant barrier to improving clinical outcomes, highlighting the urgent need for alternative therapeutic strategies.

Signal transducer and activator of transcription 3 (STAT3) is a pivotal transcription factor that mediates cellular responses to extracellular signals, particularly those involved in cell cycle regulation [10]. Aberrant activation of STAT3 has been widely observed in various cancers, including NSCLC, where it has been implicated in tumor progression, metastasis, and therapeutic resistance [11]. Upon activation by upstream kinases such as JAKs or receptor tyrosine kinases, STAT3 is phosphorylated, dimerized, and translocated to the nucleus to regulate the expression of genes critical for cell cycle progression [12]. This dysregulation enables cancer cells to bypass normal growth control mechanisms and maintain sustained proliferative signaling, a hallmark of cancer [13]. STAT3 directly regulates several genes that are essential for the G1–S phase transition, a critical checkpoint in the cell cycle [14]. Among its target genes, cyclin D1 (*CCND1*) is prominently upregulated by STAT3 activation [15]. Cyclin D1, in complex with CDK4, phosphorylates the retinoblastoma (Rb) protein, releasing E2F transcription factors to initiate the transcription of genes required for DNA replication [16]. Additionally, STAT3 suppresses the expression of cell cycle inhibitors such as p21 (*CDKN1A*) and p27 (*CDKN1B*), further tipping the balance in favor of cell cycle progression [17]. This dual role of STAT3, the induction of positive regulators and suppression of inhibitors, renders it a central player in promoting cell division in cancer cells. Beyond direct regulators of the cell cycle, STAT3 also modulates genes that support the cellular environment necessary for proliferation. STAT3-driven upregulation of c-Myc enhances the expression of metabolic and biosynthetic genes required for tumor growth [18], while the activation of anti-apoptotic genes such as *Bcl-2* ensures cellular survival during rapid proliferation [19]. Given the critical role of STAT3 in tumor growth and progression, targeting the STAT3 signaling pathway represents a promising therapeutic strategy.

In this study, we aimed to investigate the anticancer effects of the *Allium pseudojaponicum Makino* extract (APE) in human NSCLC cell lines, including cisplatin-sensitive (A549-ctr and H460-ctr) and cisplatin-resistant (A549-CR and H460-CR) cells. To evaluate the anticancer effects, we conducted a cell viability assay, colony formation assay, and soft agar assay. We further assessed the regulatory effect of APE on the cell cycle using flow cytometry and analyzed the expression of cell cycle-associated proteins such as cyclins and CDKs. To identify potential target proteins and pathways, we performed proteomic analysis and bioinformatics data processing. Additionally, the chemical composition of APE was characterized using a high-resolution liquid chromatograph-mass spectrometer (HR LC-MS). Our findings provide a foundation for further exploration of APE as a natural product-based therapeutic strategy, potentially contributing to the advancement of marine life science and technology.

## 2. Results

### 2.1. Characterization of Chemical Constituents in the Extract of Allium pseudojaponicum Makino by UPLC-MS^E^

**Figure 1:**
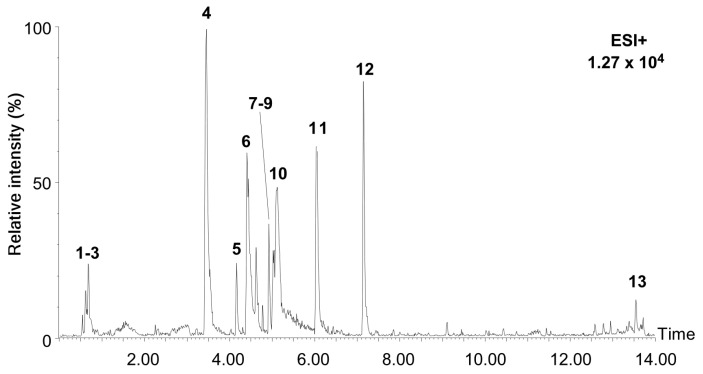
Characterization of APE by UPLC-MS^E^. Base peak intensity (BPI) chromatogram of APE in positive ion mode by UPLS-MS^E^. BPI chromatograms of 50 ug APE.

We performed metabolite profiling of *Allium pseudojaponicum Makino* extract (APE) using UPLC-Q-TOF-MS^E^. The metabolic analysis was conducted using an untargeted approach in the positive ion mode. The base peak intensity (BPI) chromatograms of APE are presented in Figure 1. To identify the detected compounds in APE by UPLC-MS^E^ analysis, the obtained data were compared with spectral libraries and compound databases, focusing on the monoisotopic mass-to-charge (*m*/*z*) of MS1 spectra and the fragment ion patterns of MS2 spectra. The results of the LC-MS chromatograms, MS1 and MS2 spectra indicated that the profiling of metabolic compounds in APE was successfully performed in the positive ion mode (Supplementary Figure S1). The identified compound adducts included [M + H]^+^, [M + Na]^+^, and [M + NH_4_]^+^. The detected and identified compounds in APE are presented in Table 1. By comparing the mass spectra of APE with the spectral libraries and compound databases available in the MS-DIAL metabolomics database library, 13 compounds were characterized and identified using retention times, molecular formula, and relative percentages. According to the findings, the identified compounds in APE were classified into several main ontological categories, with alkaloids being the most abundant (71.65%), followed by flavonoids (8.81%), flavonoid-glycosides (2.99%), O-glycosyl compounds (1.79%), pynone derivatives (1.52%), and oligosaccharides (1.03%), as determined in the positive ion mode by LC-MS analysis. Based on the peak area, the major metabolites identified were lycorine (29.81%), tazettine (17.22%), atanine (12.59%), tricetin (8.19%), ungvedine (5.49%), reserpine (3.55%), and reticuline (2.99%). Additionally, LC-MS analysis confirmed the presence of several flavonoid-glycosides, including hyperoside and rutoside, as well as oligosaccharides, including maltulose and maltotriose, in the APE. Taken together, alkaloids and flavonoids are the major compounds present in APE, both of which have traditionally been reported as bioactive compounds with anticancer properties. Therefore, this study aims to investigate the potential anticancer effects of APE.

### 2.2. APE Suppresses Proliferation and Colony Formation of Cisplatin-Resistant NSCLC Cells

**Figure 2:**
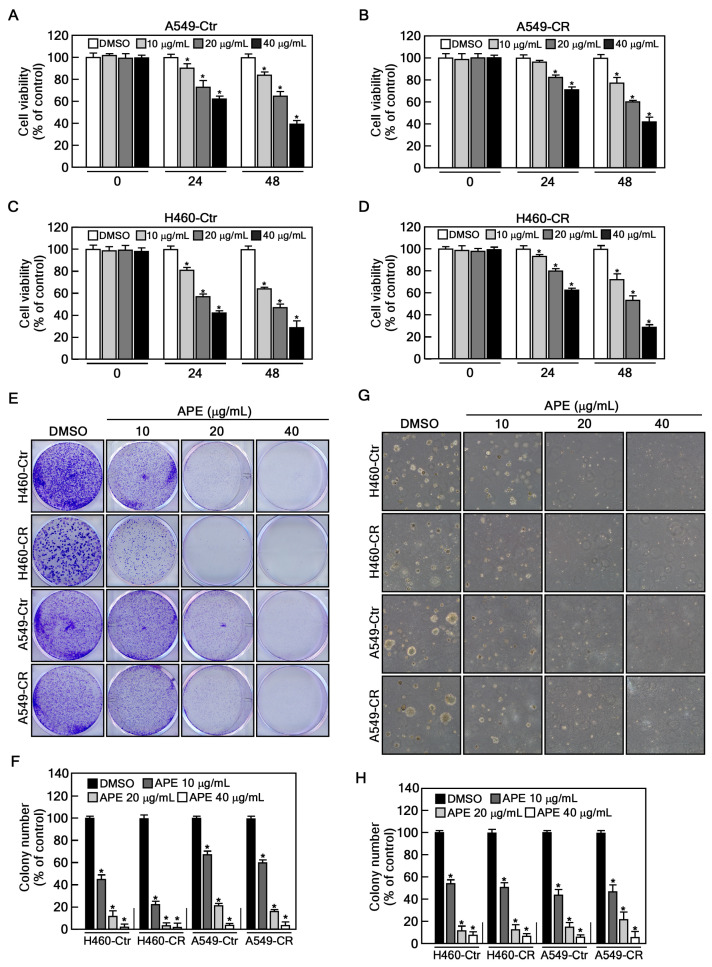
APE inhibits cisplatin-resistant NSCLC proliferation and colony growth. (**A**–**D**) Cell viability was assessed in A549-Ctr, A549-CR, H460-Ctr, and H460-CR following treatment with the indicated concentrations of APE using a CCK-8 assay. (**E**,**F**) A549-Ctr, A549-CR, H460-Ctr, and H460-CR (1 × 10^3^ cells/well) were seeded and treated with the indicated concentrations of APE. After 10 days, the medium was removed, and cells were stained with Coomassie blue and scanned using a plate reader. The number of colonies was counted using Image J software (ver. 1.54d). (**G**,**H**) A549-Ctr, A549-CR, H460-Ctr, and H460-CR cells (8 × 10^3^ cells) were suspended in BME medium containing 0.3% agar supplemented with 10% FBS and treated with vehicle (DMSO), 10, 20, or 40 µg/mL of APE. Cultures were maintained at 37 °C in a 5% CO_2_ incubator for 20 days. Data were obtained from three independent experiments. Statistical significance was determined as follows: * *p* < 0.05.

Before evaluating the anticancer effect of APE in cisplatin-sensitive and cisplatin-resistant NSCLC cells (A549 and H460), we first confirmed cisplatin resistance in these cells. Cells were treated with increasing concentrations of cisplatin (0, 1.25, 2.5, 5, and 10 μM) for 48 h. The results showed that cisplatin significantly decreased the viability of the of cisplatin-sensitive cells (A549-Ctr and H460-Ctr) in a dose-dependent manner. In contrast, cisplatin-resistant cells (A549-CR and H460-CR) exhibited sustained viability under the same conditions, indicating their resistance to cisplatin (Supplementary Figure S2A,B). Next, we evaluated the anti-proliferative effect of APE on cisplatin-sensitive or cisplatin-resistant cells using a cell viability assay. APE treatment led to a dose- and time-dependent reduction in viability in both cisplatin-sensitive and -resistant cells. At 48 h, treatment with 40 µg/mL APE reduced cell viability to 38.6% in A549-Ctr, 37.2% in A549-CR, 28.4% in H460-Ctr, and 30.4% in H460-CR, compared to the control group (Figure 2A–D). Notably, APE did not exhibit cytotoxicity in HaCaT cells, a human keratinocyte cell line, even at concentration up to 80 μg/mL (Supplementary Figure S2C). To further confirm the anticancer effects of APE, we conducted a colony formation and an anchorage-independent soft agar assay, both widely used in vitro techniques to assess the ability of a single cell to survive, proliferate, and form a colony. In 2D colony formation assays, APE treatment led to a substantial reduction in colony numbers, particularly at 40 µg/mL, where colony numbers decreased to 5.8% (H460-Ctr), 3.3% (H460-CR), 6.1% (A549-Ctr), and 6.7% (A549-CR) of control levels (Figure 2E,F). Similarly, in soft agar assays, colony formation was markedly inhibited, with more than a 90% reduction at 40 µg/mL APE (Figure 2G,H). Collectively, these findings indicate that APE effectively inhibits cell proliferation and colony formation in cisplatin-resistant NSCLC cells.

### 2.3. APE Attenuates Cell Cycle Progression at the G1 Phase

**Figure 3:**
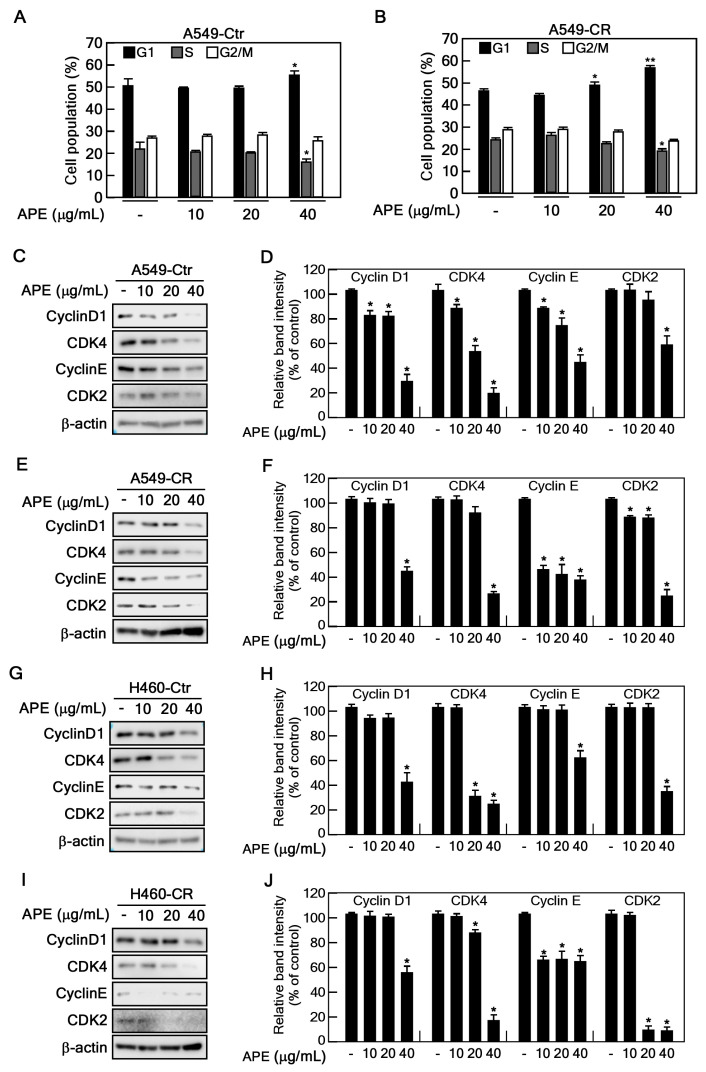
APE induces cell cycle arrest in the G1 phase. (**A**,**B**) A549-Ctr and A549-CR cells (3 × 10^5^ cells) were seeded into 60 mm dishes. The next day, cells were treated with the indicated concentrations of APE for 24 h. After detachment, cells were suspended in 0.6% Triton X-100/PBS and incubated with 200 µg/mL RNase A and 20 µg/mL propidium iodide for 15 min at 4 °C. Cell cycle phases were analyzed by flow cytometry. (**C**–**J**) A549-Ctr, A549-CR, H460-Ctr, and H460-CR cells (5 × 10^5^ cells) were seeded into 100 mm cell culture dishes. Cells were treated with vehicle (DMSO) or APE at concentrations of 10, 20, or 40 µg/mL for 24 h. Proteins were extracted and analyzed by Western blotting using specific antibodies as indicated. Protein expression levels were normalized to β-actin. Statistical significance was determined as follows: * *p* < 0.05, ** *p* < 0.01.

Given that APE reduces cell proliferation and colony formation in cisplatin-resistant NSCLC cells, we performed flow cytometry analysis to evaluate its effects on cell cycle distribution. Treatment with APE significantly increased the proportion of cells in the G1 phase while reducing the proportion in the S phase in both A549-Ctr and A549-CR cells, indicating that APE induces cell cycle arrest at the G1 phase (Figure 3A,B). Specifically, in A549-Ctr cells the G1 population increased from 50.4% (control) to 56.6% at 40 µg/mL APE, accompanied by a decrease in the S phase from 22.6% to 13.3%. Similarly, in A549-CR cells the G1 population rose from 47.5% to 58.4%, while the S phase decreased from 24.3% to 18.4% at the same concentration. Consistent with the cell cycle analysis, we next examined the expression of key regulatory proteins involved in the G1–S phase transition, including cyclin D1, CDK4, cyclin E, and CDK2, using Western blot analysis. We found that APE markedly downregulated the expression of cyclin D1, CDK4, cyclin E, and CDK2 in A549-Ctr, A549-CR, H460-Ctr, and H460-CR cells (Figure 3C–J). Collectively, these results suggest that APE suppresses cell proliferation by inducing G1 phase arrest via the downregulation of G1–S phase transition proteins.

### 2.4. APE Targets STAT3 Signaling Pathway

**Figure 4:**
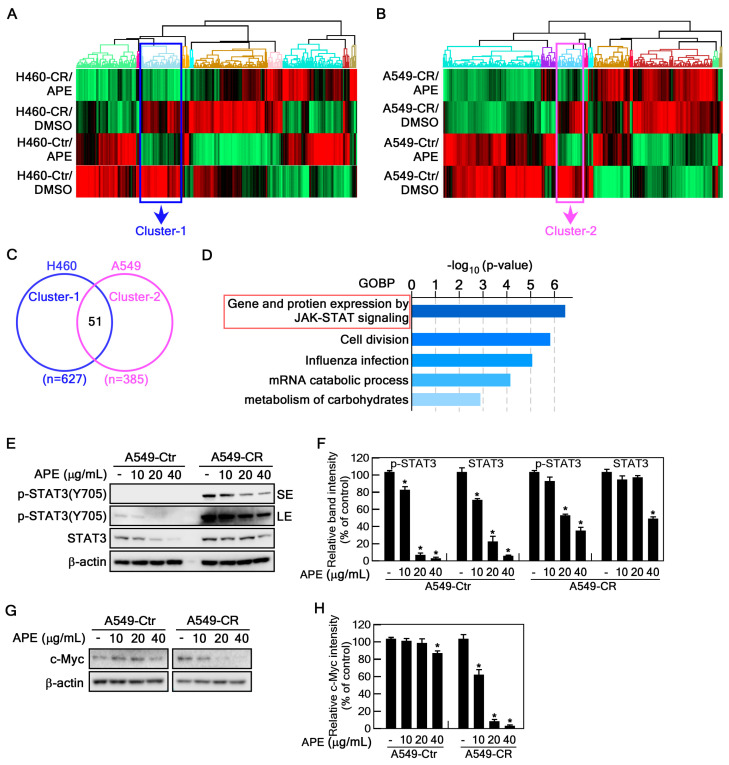
Identification of the APE target signaling pathway. (**A**,**B**) Heat maps showing differentially expressed genes in H460 (**A**) and A549 (**B**) cells. Control (Ctr) and CR cells were treated with either DMSO or 20 μg/mL of APE. Upregulated genes are indicated in red, and downregulated genes are indicated in green. The sample conditions are labeled at the top of each heat map. In total, 627 genes were significantly altered in H460 cells, while 385 genes were significantly altered in A549 cells. (**C**) A Venn diagram illustrating the overlap of significantly altered genes in H460-Ctr, H460-CR, A549-Ctr, and A549-CR cells. Fifty-one genes were commonly changed across all four groups. (**D**) GO biological process (GOBP) analysis of the overlapping genes was performed, and the results are presented in order of -log10 (*p*-value). The top enriched processes include JAK-STAT-mediated protein expression, cell division, influenza infection, mRNA catabolic processes, and carbohydrate metabolism. (**E**–**H**) A549-Ctr and A549-CR (5 × 10^5^ cells) cells were seeded into 100 mm cell culture dishes, cultured for 24 h. APE was treated with vehicle (DMSO), 10, 20, or 40 µg/mL for 24 h. SE; short exposure, LE; long exposure. The proteins were extracted and visualized by Western blotting using specific antibodies as indicated. Protein expression levels were normalized to β-actin. Statistical significance was determined as follows: * *p* < 0.05.

To identify the potential signaling pathway targeted by APE, we performed proteomic analysis on H460-Ctr, H460-CR, A549-Ctr, and A549-CR with and without APE treatment. Heatmap analysis revealed significant changes in protein expression following APE treatment, with upregulated proteins shown in red and downregulated proteins in green. Notably, distinct expression patterns were observed between control and cisplatin-resistant cell variants (Figure 4A,B). The differentially expressed proteins were categorized into 10 clusters, and our analysis focused on clusters of proteins downregulated by APE treatment, specifically cluster 1 (627 proteins) in H460 cells and cluster 2 (385 proteins) in A549 cells (Supplementary DEP list). Venn diagram analysis identified 51 overlapping proteins between these two clusters (Figure 4C). Next, we conducted gene ontology biological process (GOBP) enrichment analysis on these 51 proteins. The results revealed significant enrichment in biological processes including JAK-STAT signaling, cell division, influenza infection, mRNA catabolic processes, and carbohydrate metabolism (Figure 4D). Among these, the JAK-STAT signaling pathway exhibited the highest level of enrichment, suggesting its critical role in mediating the cellular response to APE treatment. Given the well-established role of the STAT signaling pathway, particularly STAT3, in cell growth, division, and oncogenesis, we hypothesized that APE might target the STAT3 signaling pathway. To investigate this hypothesis, we analyzed STAT3 protein expression and phosphorylation levels in APE-treated A549-Ctr and A549-CR cells. APE treatment significantly reduced STAT3 phosphorylation at Tyr705, as well as its total protein levels (Figure 4E,F). To further confirm that APE targets STAT3, we performed Western blot analysis of STAT3 downstream effectors, such as c-Myc [28]. APE treatment markedly inhibited c-Myc expression in a dose-dependent manner (Figure 4G,H). Together, these results indicate that APE effectively targets the STAT3 signaling pathway.

### 2.5. STAT3 Depletion Showed SIMILAR Anticancer Effect of APE

**Figure 5:**
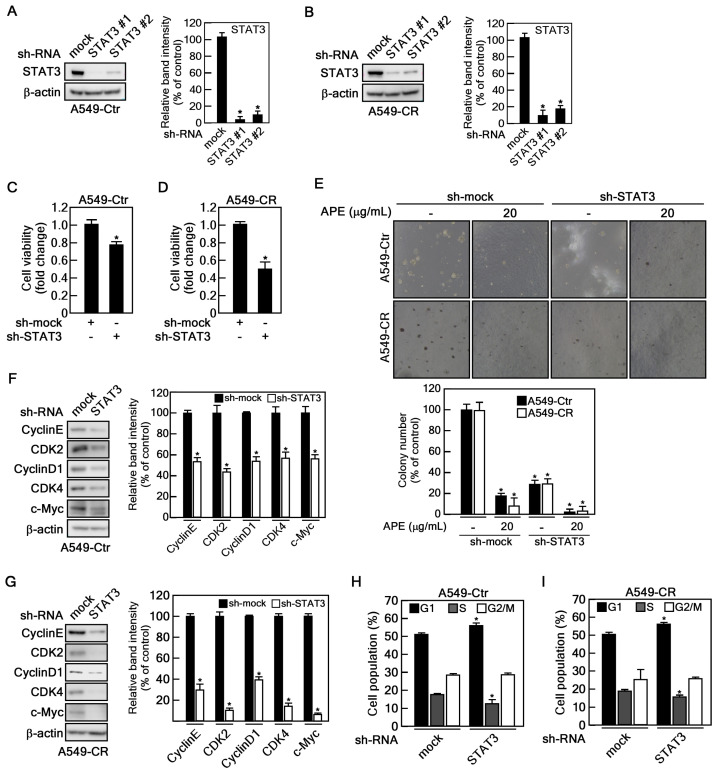
STAT3 depletion exhibited an anticancer effect similar to that of APE. (**A**,**B**) Generation of A549-Ctr and A549-CR with STAT3 knockdown using two different small hairpin RNAs that target STAT3. (**C**,**D**) A549-Ctr and A549-CR cells with STAT3 knockdown (3 × 10^3^ cells/well) were seeded into 96-well plates and cultured. Cell viability was assessed using a CCK-8 assay. (**E**) Inhibition of anchorage-independent colony growth of A549-Ctr, A549-CR, H460-Ctr, and H460-CR with STAT3 knockdown cells (8 × 10^3^ cells/well) were suspended in BME medium containing 0.3% agar supplemented with 10% FBS and 20 µg/mL of APE. Cultures were maintained at 37 °C in a 5% CO_2_ incubator for 20 days. Colony numbers were counted and quantified using an ECLIPSE Ti inverted microscope and the NIS-Elements AR (V. 4.0) software program (NIKON Instruments Korea). (**F**,**G**) A549-Ctr, A549-CR, H460-Ctr, and H460-CR with STAT3 knockdown cells (5 × 10^5^ cells) were seeded into 100 mm cell culture dishes and cultured for 24 h. Proteins were extracted and analyzed by Western blotting using specific antibodies as indicated. Protein expression levels were normalized to β-actin. Statistical significance was determined as follows: * *p* < 0.05. (**H**,**I**) A549-Ctr and A549-CR cells with STAT3 knockdown (3 × 10^5^ cells/well) were seeded into 60 mm dishes and cultured for 24 h. After detaching the cells, they were suspended in 0.6% Triton X-100/PBS and incubated with 200 µg/mL RNase A and 20 µg/mL propidium iodide for 15 min at 4 °C. The cell cycle phases were analyzed by flow cytometry.

As described above, APE treatment inhibits cell proliferation, colony formation, STAT3 phosphorylation, and total STAT3 protein levels in cisplatin-resistant NSCLC cells (Figure 2 and Figure 4). To investigate whether STAT3 mediates cisplatin resistance similar to APE treatment, we established STAT3 knockdown in A549-Ctr and A549-CR cells using two distinct small hairpin RNAs (shRNAs) targeting STAT3 (Figure 5A,B). STAT3 depletion significantly suppressed cell proliferation in both A549-Ctr and A549-CR cells, with a more pronounced reduction in viability in A549-CR cells (Figure 5C,D). Furthermore, STAT3 knockdown abrogated colony formation, and APE treatment of STAT3-depleted cells further enhanced this inhibition (Figure 5E). Additionally, STAT3 knockdown significantly reduced the expression of key cell cycle regulatory proteins, including cyclin E, CDK2, cyclin D1, and CDK4, as well as its downstream target, c-Myc (Figure 5F,G). Consistent with these findings, STAT3 depletion induced cell cycle arrest at the G1 phase in both A549-Ctr and A549-CR cells (Figure 5H,I). These findings suggest that STAT3 plays a critical role in mediating cisplatin resistance and that APE is an effective inhibitor of the STAT3 signaling pathway.

## 3. Discussion

Prior to this study, we conducted a preliminary screening of 50 halophyte extracts for their anticancer properties in lung cancer cells. Among these, *Allium pseudojaponicum Makino* exhibited the most potent anticancer activity, prompting its selection for further investigation. In this study, we demonstrated that *A. pseudojaponicum* extract (APE) effectively suppressed the proliferation and colony-forming ability of both cisplatin-sensitive and cisplatin-resistant NSCLC cells by inducing G1 phase cell cycle arrest. Through proteome analysis, we identified the STAT3 signaling pathway as a critical target of APE. Furthermore, LC-MS analysis confirmed the presence of key bioactive compounds, including alkaloids and flavonoids, which may contribute to its anticancer effects.

One of the most notable findings of this study is that APE exhibits significant anticancer effects in cisplatin-resistant NSCLC cells (Figure 2). Cisplatin resistance is a major clinical hurdle that limits treatment efficacy and leads to a poor prognosis in lung cancer patients [29,30]. Our results show that APE reduces the viability of both cisplatin-sensitive (A549-Ctr, H460-Ctr) and cisplatin-resistant (A549-CR, H460-CR) cells without cytotoxicity in normal cells, suggesting that APE may help overcome cisplatin resistance mechanisms. In particular, APE nearly abolished colony formation in both 2D and 3D conditions, indicating its potent anti-proliferative effects. These findings suggest that APE could be a promising therapeutic candidate, either as a standalone treatment or in combination with standard chemotherapy, to enhance treatment outcomes in cisplatin-resistant NSCLC.

Uncontrolled cell cycle progression is a hallmark of cancer, allowing tumor cells to proliferate indefinitely [31]. We found that APE significantly increased the proportion of cells in the G1 phase while reducing the S phase population, suggesting that it induces G1 phase arrest to inhibit cancer cell growth (Figure 3). This effect was further confirmed by the downregulation of key G1–S transition regulators, including cyclin D1, CDK4, cyclin E, and CDK2. These proteins play essential roles in driving cell cycle progression [32], and their suppression by APE suggests that it effectively halts the proliferation of NSCLC cells by preventing their entry into the S phase. Given that dysregulated cell cycle control is a common feature of cancer, the ability of APE to induce G1 arrest provides a strong mechanistic rationale for its anti-proliferative effects.

An important mechanistic insight from this study is that APE directly targets the STAT3 signaling pathway (Figure 4), which plays a crucial role in promoting tumor progression, survival, and cisplatin resistance [33,34]. Proteomic analysis revealed that STAT3 was one of the most significantly downregulated proteins following APE treatment. Western blot analysis further confirmed that APE reduces both STAT3 phosphorylation at Tyr705 and total STAT3 protein levels, leading to the suppression of downstream targets such as c-Myc, a key oncogene involved in tumor growth. STAT3 activation is frequently associated with poor prognosis and drug resistance in NSCLC. Its inhibition has been proposed as a therapeutic strategy to restore chemotherapy sensitivity [35]. While several synthetic STAT3 inhibitors (e.g., Stattic, WP1066) are under investigation, they often suffer from low bioavailability and off-target toxicity [36]. Unlike synthetic inhibitors, APE, a natural extract, may exert anticancer effects through multi-targeted interactions, potentially improving therapeutic selectivity and reducing toxicity. The observation that STAT3 knockdown mimics the effects of APE treatment further supports the hypothesis that APE exerts its anticancer activity primarily through STAT3 inhibition.

Our findings align with previous reports demonstrating the anticancer effects of plant-derived compounds through STAT3 inhibition and cell cycle arrest. Natural compounds such as curcumin, resveratrol, and epigallocatechin gallate (EGCG) have been shown to downregulate STAT3 signaling in lung and other cancers [37,38,39]. However, APE contains a diverse array of bioactive metabolites, including alkaloids (lycorine, tazettine) and flavonoids (tricetin, rutoside), which may confer broader and potentially more potent anticancer effects compared to single-compound therapies (Table 1). Moreover, previous research has confirmed that lycorine, a major component of APE (29.81%), directly binds to STAT3 and suppresses colorectal cancer cell growth [40]. These findings highlight the potential of APE as a novel natural inhibitor of STAT3, with implications for overcoming drug resistance in lung cancer therapy. Notably, cisplatin, one of the most widely used chemotherapeutic agents for NSCLC, exhibits IC₅₀ values ranging from 3 to 10 µM in cisplatin-sensitive NSCLC cell lines, while showing markedly reduced efficacy in resistant models, with viability often exceeding 70% [9]. In our study, treatment with 40 µg/mL APE reduced cell viability to 28.4–38.6% in both sensitive and resistant NSCLC cells. This suggests that APE maintains its efficacy regardless of cisplatin resistance, unlike cisplatin itself. In addition, whereas cisplatin induces apoptosis primarily through DNA damage, APE inhibits cancer cell growth via a distinct mechanism involving G1 phase cell cycle arrest and suppression of the STAT3 signaling pathway. This complementary mechanism highlights APE’s potential to overcome resistance and support combination therapy strategies. Moreover, APE demonstrated minimal toxicity to normal HaCaT cells up to 80 µg/mL, in contrast to the well-documented nephro- and neurotoxicity of cisplatin, further supporting its therapeutic selectivity.

Although we provide compelling evidence of the anticancer effects of APE, this study has two limitations. First, while APE demonstrated strong anticancer activity against both cisplatin-sensitive and cisplatin-resistant NSCLC cells, its efficacy, pharmacokinetics, and safety profile in animal models remain to be determined. Second, while metabolite profiling identified several major bioactive compounds, including alkaloids and flavonoids, it remains unclear whether the anticancer effects of APE are mediated by a single active compound or the synergistic interaction of multiple components. Thus, further studies are needed to evaluate the therapeutic potential of APE in in vivo models and to isolate and characterize its most potent bioactive molecules.

**Figure 6:**
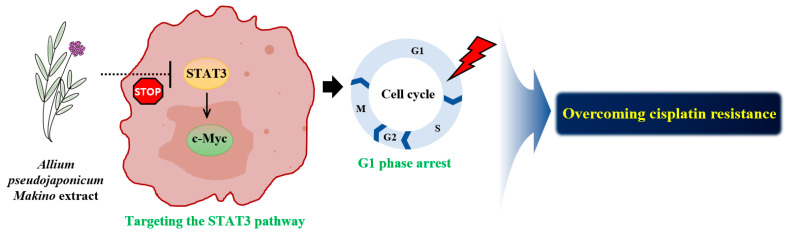
Schematic summary of the proposed mechanism. A graphical summary illustrating the working model of how APE targets the STAT3 signaling pathway and induces G1 phase arrest to overcome cisplatin resistance in NSCLC.

In conclusion, this study provides strong evidence that APE is a promising natural compound with potent anticancer activity against NSCLC, particularly in overcoming cisplatin resistance. By modulating critical oncogenic pathways, inducing G1 phase arrest, and inhibiting STAT3 signaling pathway, APE effectively suppresses cancer cell proliferation and colony formation. Therefore, these findings suggest that APE may serve as a novel therapeutic strategy, either as a standalone treatment or in combination with current chemotherapies for cisplatin-resistant NSCLC (Figure 6).

## 4. Materials and Methods

### 4.1. Extract of Allium Pseudojaponicum Makino (APE)

APE (MABIK NP60210017) was obtained from the National Marine Biodiversity Institute of Korea (MABIK). The *A. pseudojaponicum Makino* was collected on 10 April 2018, in Pyoseon-myeon, Seogwipo-si, Jeju Special Self-Governing Province, South Korea. To prepare the extract, *A. pseudojaponicum Makino* specimens were washed three times with tap water and freeze-dried using an OPERON FDT-8650 freeze dryer. The dried material (30 g) was ground into a fine powder and suspended in 400 mL of 70% ethanol. Extraction was performed three times at room temperature using an ultrasonic extractor (DAIHAN WUC-N30H) set at 40 kHz for 60 min per cycle. The obtained extract was filtered through Whatman 2V filter paper (320 mm) and concentrated using rotary evaporation (Buchi CH/R-210).

### 4.2. Reagents

Dimethyl sulfoxide (Cat #: D2650) was purchased from Sigma–Aldrich (Sigma–Aldrich Korea, Gangnam, Seoul, Republic of Korea). Antibodies including STAT3 (Cat #: 9139), p-STAT3(Y705) (Cat#: 9145), cyclin E (Cat #: 4129), and CDK4 (Cat #: 12790) were acquired from Cell Signaling Technology (Danvers, MA, USA). CDK2 (Cat #: sc-6248), c-Myc (Cat #: sc-788), and β-actin (Cat #: sc-47778) were purchased from Santa Cruz Biotechnology (Dallas, TX, USA). Cyclin D1 (Cat #: ac134175) was supplied by Abcam (Abcam Korea, Hanam-si, Gyeonggi-do, Republic of Korea). Cisplatin was purchased from Ildong (Ildong pharm. Co., Ltd., Seoul, Republic of Korea). Coomassie Blue R-250 (Cat #: CBC006) for colony formation assay was purchased from LPS solution (Daejeon-si, Republic of Korea). RNase A (Cat #: 70856) was purchased from Millipore (Burlington, MA, USA) and propidium iodide (Cat #: P4170) was purchased from Sigma-Aldrich (St. Louis, MI, USA) for flow cytometry.

### 4.3. Cell Culture and Treatment

The A549 and H460 NSCLC cell lines, including their control (A549-Ctr and H460-Ctr) and cisplatin-resistant (A549-CR and H460-CR) variants, were provided by Prof. Jin Kyung Rho [41]. HEK293T cells were obtained from ATCC. The NSCLC cells were cultured in RPMI1640 supplemented with 10% fetal bovine serum (FBS) and 1% penicillin/streptomycin. HEK293T cells were cultured in DMEM supplemented with 10% fetal bovine serum (FBS) and 1% penicillin/streptomycin. All cells used in this research were maintained in a humidified atmosphere with 5% CO_2_. To prepare the treatment solution, APE was dissolved in dimethyl sulfoxide (DMSO) and administered at the specified concentration and incubation time.

### 4.4. Cell Viability Assay

To validate the impact of APE on cell viability, A549-Ctr, A549-CR, H460-Ctr, and H460-CR cells were plated in 96-well plates at a density of 1 × 10^3^ cells/well and allowed to pre-incubate for 24 h. Cells were then treated with APE at final concentrations of 10, 20, and 40 µg/mL for either 24 or 48 h. Control wells received RPMI1640 medium containing 0.1% (*v*/*v*) DMSO. Cell viability was determined using the cell counting kit-8 (CCK-8, Dojindo, Japan) solution. After the addition of 10 µL of CCK8 solution, cells were incubated at 37 °C in a 5% CO_2_ humidified atmosphere for 1 h. Absorbance at 450 nm was measured using a microplate reader (Tecan, Männedorf, Switzerland).

### 4.5. Western Blotting

Cells were lysed using an EBC buffer (50 mM Tris-HCl, 120 mM NaCl, and 0.5% Nonidet P-40 Substitute) supplemented with protease and phosphatase inhibitors. The lysate concentration was determined using the Bradford reagent (MP200, Biosolution, Republic of Korea). Equal amounts of protein were separated via 8–10% SDS-PAGE and transferred onto a PVDF membrane (Immobilon-P, Millipore). After washing with TBST, the membrane was blocked with 5% skimmed milk at room temperature for 1 h. Primary antibody incubation was performed at 4 °C overnight, followed by secondary antibody incubation at room temperature for 1 h. Protein detection was carried out using the ECL detection system (Advansta) and imaged with the Azure 280 chemiluminescent imaging system (Azure Biosystems, Dublin, CA, USA). The band intensity was quantified using ImageJ (ver. 1.54d), normalized to β-actin, and analyzed via Tukey’s multiple comparison test. A *p*-value < 0.05 was considered statistically significant.

### 4.6. Gene Silencing

Endogenous STAT3 was depleted using shRNA vectors (#TRCN0000020840, TRCN0000020843) obtained from Sigma. To generate viral particles containing sh-STAT3, HEK293T cells were transfected with sh-STAT3 along with packaging plasmids (pMD2.G and psPAX2). After incubation for 48 h, the culture medium containing viral particles was collected and filtered through 0.4 μm syringe filters. The viral particles were then used to infect A549 and A549-CR cells, with 2 μg/mL polybrene (#TR-1003-G, Millipore, MA, USA) added to enhance infection efficiency. Following infection, cells were selected with 10 μg/mL puromycin (#A11138, Thermo Fisher Scientific, Waltham, MA, USA) for 2 days to ensure that only infected cells remained.

### 4.7. Anchorage-Independent Colony Growth Assay (Soft Agar Assay)

To assess the anchorage-independent growth ability of cancer cells, a soft agar colony formation assay was performed. A bottom layer was prepared by mixing 1.2% agarose with complete BME medium (containing 10% fetal bovine serum and 1% penicillin-streptomycin) at a 1:1 ratio, yielding a final agarose concentration of 0.6%. This mixture was poured into 6-well plates and allowed to solidify at room temperature. A top agar layer containing 0.3% agarose and a suspension of 8 × 10^3^ cells/well in complete BME medium was then added above the base layer. The indicated concentrations of APE were added to both the bottom and top agar layers. Plates were incubated at 37 °C in a 5% CO_2_ humidified atmosphere for 20 days to allow colony formation. Colony numbers were counted and quantified using an ECLIPSE Ti inverted microscope and the NIS-Elements AR (v4.0) software program (NIKON Instruments, Seoul, Republic of Korea).

### 4.8. Cell Cycle Analysis

A549-Ctr and A549-CR (3 × 10^5^) cells were seeded into 60 mm dishes and cultured overnight at 37 °C in a 5% CO_2_ incubator. Cells were treated with either the vehicle (DMSO) or APE at concentrations of 10, 20 and 40 µg/mL for 24 h, then trypsinized, washed with ice-cold 1X PBS, and fixed with ice-cold 70% ethanol. The fixed cells were suspended in 0.6% Triton X-100/PBS and treated with 200 µg/mL RNase A and 20 µg/mL propidium iodide for 15 min at 4 °C. The cell cycle phase distribution was analyzed by using flow cytometry (Invitrogen Attune NxT Flow Cytometry, Waltham, MA, USA).

### 4.9. S-Trap-Based Protein Digestion

Protein lysates, containing 150 ug per sample, were initially solubilized in an SDS-containing lysis buffer (e.g., 5% SDS in 50 mM TEAB) to ensure complete protein denaturation. The samples were then reduced using 10 mM tris(2-carboxyethyl) phosphine (TCEP) at 56 °C for 30 min and subsequently alkylated with 20 mM iodoacetamide (IAA) at room temperature in the dark for 30 min. The acidified lysates containing 1.2% phosphoric acid were diluted sevenfold with a binding buffer consisting of 90% methanol in 100 mM TEAB and loaded onto an S-Trap mini column (Protifi, NY, USA) by centrifugation at 4000× *g* for 1 min. The proteins were washed three times with binding buffer to remove contaminants. For digestion, 10 µg of sequencing-grade trypsin was added to the columns in 50 mM TEAB, and the samples were incubated overnight at 37 °C. Peptides were eluted sequentially using 50 mM TEAB, followed by 0.1% formic acid, and then 50% acetonitrile in 0.1% formic acid. The eluates were pooled and dried under a vacuum for further analysis.

### 4.10. TMT Labeling and Fractionation

Tryptic peptides were labeled using the TMT 10-plex isobaric labeling reagent kit (Thermo Fisher Scientific, Waltham, MA, USA) following the manufacturer’s protocol. Each sample was labeled with a unique TMT tag in 100 mM TEAB buffer at room temperature for 1 h. Labeling reactions were quenched with 5% hydroxylamine for 15 min, and the labeled peptides were pooled into a single tube. The pooled samples were fractionated using the high-pH reversed-phase peptide fractionation kit (Thermo Fisher Scientific, Waltham, MA, USA) according to the manufacturer’s instructions. Fractionation yielded 10 fractions, which were dried under a vacuum for subsequent analysis.

### 4.11. LC-MS/MS Analysis

Peptide analysis was conducted using an LC-MS/MS system comprising a Dionex UltiMate 3000 RSLCnano system and an Orbitrap Eclipse Tribrid mass spectrometer (Thermo Fisher Scientific, Waltham, MA, USA). Peptides were initially loaded onto a trap column (75 µm × 2 cm, C18, nanoViper, Acclaim PepMap100, Thermo Fisher Scientific) for pre-concentration and desalting. Subsequently, separation was performed on an analytical column (75 µm × 50 cm, C18, PepMap RSLC, Thermo Fisher Scientific) at a flow rate of 250 nL/min. The mobile phases consisted of 0.1% formic acid in water (mobile phase A) and 0.1% formic acid in acetonitrile (mobile phase B). Throughout the chromatographic separation, the Orbitrap Eclipse Tribrid mass spectrometer was operated in data-dependent acquisition (DDA) mode. MS1 spectra were acquired in the Orbitrap analyzer with a mass range of 400–2000 *m*/*z*, and a resolution of 120,000. For MS2 spectra, the Orbitrap mass analyzer operated at a resolution of 30,000 using turbo-TMT settings with high-energy collision dissociation (HCD) at a normalized collision energy of 36%.

### 4.12. Protein Identification and Quantification

Raw data files generated from LC-MS/MS were processed using IP2 software (v6.5.5; Integrated Proteomics Applications, Inc., San Diego, CA, USA) for protein and peptide identification. Peak lists were generated and searched against the UniProt human protein database, with the following search parameters: carbamidomethylation of cysteine as a fixed modification, oxidation of methionine as a variable modification, and allowance for up to two missed tryptic cleavages. The search engine used a mass tolerance of ±10 ppm for precursor ions and ±50 ppm for fragment ions. Search results were filtered to a false discovery rate (FDR) of less than 1%.

### 4.13. Bioinformatics Analysis

Differentially expressed proteins were analyzed using Metascape, a comprehensive online tool for functional annotation and enrichment analysis. Proteins were annotated with gene ontology (GO) terms, including biological processes, molecular functions, and cellular components categories.

### 4.14. Metabolomic Analysis Using UPLC-QTOF-MS^E^

UPLC-QTOF-MS analysis was performed using a Waters ACQUITY UPLC system equipped with a Waters Synapt G2 HDMS (Waters, Milford, MA, USA) and operated under MassLynx 4.1 software. Chromatographic separation was conducted on a Waters ACQUITY UPLC HSS T3 column (100 × 2.1 mm, 1.8 μm). The column temperature was set to 35 °C, and the sample chamber temperature was maintained at 10 °C. A binary mobile phase was used, consisting of 0.1% formic acid (phase A) and acetonitrile (phase B), with the following gradient: 0–1 min, 2% B; 1–8 min, 2–50% B; 8–10 min, 50–95% B; 10–13 min, 95% B; 13–13.1 min, 95–2% B; 13.1–15 min, 2% B. The flow rate was 0.4 mL·min^−1^, and the injection volume was 10 μL. The ESI source was scanned in the MSE mode under positive ionization. The optimal settings were as follows: acquisition time, 14 min; ion scanning range, 50–1200 Da; low collision energy, 6 V; high collision energy, 10–45 V; capillary voltage, 3 kV (positive mode); sample cone voltage, 40 V; extraction voltage, 4.0 V; ion source temperature, 120 °C; desolvation temperature, 450 °C; cone gas flow rate, 50 L·h^−1^; desolvation gas flow rate, 800 L·h^−1^.

### 4.15. Statistical Analysis

All experiments were repeated at least three times. Statistical analysis was performed using GraphPad Prism software (ver.10.0.2; GraphPad, La Jolla, CA, USA). Data were analyzed using Tukey’s multiple comparison test and are expressed as the mean ± standard deviation of three independent experiments. A *p*-value of less than 0.05 was considered statistically significant.

## Figures and Tables

**Table 1:** The constituents detected in APE by UPLC-Q-TOF-MS^E^. The table presents the peak numbers, retention time (RT, min), MS1 *m*/*z* values ([M + H]^+^, [M + Na]^+^, and [M + NH_4_]^+^), identification, molecular formula, ontology, and MS/MS fragment ion *m*/*z* values. The peak area was calculated separately for identified compounds detected in positive ion mode.

No.	RT (min)	MS1 (*m*/*z*)	Δppm	Molecular Formula	Identification	Ontology	MS/MS (*m*/*z*)	Peak Area (%)	Ref
1	0.66	365.1046	2.44	C_12_H_22_O_11_	Isomaltulose	O-glycosyl compounds	85.03, 127.04, 145.05, 163.06	1.79	[20]
2	0.70	127.0383	5.35	C_6_H_6_O_3_	Triacetate lactone	Pynone derivatives	69.03, 85.03, 97.03, 99.04, 109.03	1.52	[21]
3	0.70	522.2040	2.22	C_18_H_32_O_16_	Melezitose	Oligosaccharides	97.03, 127.04, 145.05, 163.06, 289.09, 487.16	1.03	[22]
4	3.47	288.1241	1.74	C_16_H_17_NO_4_	Lycorine	Alkaloids	119.05, 147.04, 177.05, 194.10, 222.09, 252.10, 270.11	29.81	[23]
5	4.19	348.1817	1.64	C_19_H_25_NO_5_	Ungvedine	Alkaloids	206.93, 175.00, 141.97, 121.90	5.49	[23]
6	4.44	332.1486	3.55	C_18_H_21_NO_5_	Tazettine	Alkaloids	181.06, 211.07, 282.11, 314.14	17.22	[23]
7	4.94	303.0497	0.53	C_15_H_10_O_7_	Tricetin	Flavonoids	137.02, 149.02, 153.01, 229.05, 257.04	8.19	[24]
8	4.94	465.1018	2.09	C_21_H_20_O_12_	Hyperoside	Flavonoid-glycosides	137.02, 149.02, 153.01, 229.05, 257.04, 303.05	1.63	[24]
9	4.94	611.1630	3.89	C_27_H_30_O_16_	Rutoside	Flavonoid-glycosides	257.05, 273.03, 303.05, 465.10	1.36	[24]
10	5.34	317.0629	8.64	C_16_H_12_O_7_	3-Methylquercetin	Flavonoids	221.09, 272.13, 302.13, 314.15	0.62	[24]
11	6.07	362.1973	1.21	C_20_H_27_NO_5_	Phalaenopsine T	Alkaloids	211.08, 266.08, 302.18, 330.17	2.99	[25]
12	7.17	244.1327	4.34	C_15_H_17_NO_2_	Atanine	Alkaloids	78.03, 104.05, 156.08, 182.10, 184.07	12.59	[26]
13	13.55	609.2728	12.92	C_33_H_40_N_2_O_9_	Reserpine	Alkaloids	182.10, 196.11, 330.17, 544.27, 593.28	3.55	[27]

## Data Availability

Data will be made available upon request to the corresponding author.

## Supplementary Materials

The following supporting information can be downloaded at: https://www.mdpi.com/article/10.3390/md23040167/s1, Figure S1: Metabolic profiling of APE by UPLC/MS^E^ in positive ion mode. Figure S2: Confirmation of cisplatin resistance in NSCLC cells and cytotoxicity of APE in HaCaT cells. Metabolic profiling of APE by UPLC/MS^E^ in positive ion mode.

## Abbreviations

The following abbreviations are used in this manuscript:

APE	Extracts of *Allium pseudojaponicum Makino*
ATCC	American type culture collection
BME	Basal media eagle
BPI	Base peak intensity
BSA	Bovine serum albumin
CCK-8	Cell counting kit-8
CDK2	Cyclin-dependent kinase 2
CDK4	Cyclin-dependent kinase 4
DDA	Data-dependent acquisition
DMSO	Dimethyl sulfoxide
EBC	Eukaryotic buffer for cytoplasm
ECL	Enhanced chemiluminescence
FBS	Fetal bovine serum
FDR	False discovery rate
GOBP	Gene ontology biological process
HCD	High-energy collision dissociation
HR LC-MS	High-resolution liquid chromatograph-mass spectrometer
IAA	Iodoacetamide
JAK	Janus kinase
NSCLC	Non-small-cell lung cancer
PBS	Phosphate-buffered saline
PVDF	Polyvinylidene difluoride
SDS-PAGE	Sodium dodecyl sulfate-polyacrylamide gel electrophoresis
STAT3	Signal transducer and activator of transcription 3
TBST	Tris-Buffered Saline with Tween-20 detergent
TCEP	Tris(2-carboxyethyl) phosphine
TEAB	Triethylammonium bicarbonate
TMT	Tandem mass tag
UPLC-QTOF-MS	Ultra-high performance liquid chromatography with quadrupole time-of-flight mass spectrometry
