# Supplementary material for: Extracts from Allium pseudojaponicum Makino Target STAT3 Signaling Pathway to Overcome Cisplatin Resistance in Lung Cancer
Source: Mar Drugs. 2025 Apr 14;23(4):167. doi: 10.3390/md23040167 (PMC12028371; doi:10.3390/md23040167)
Supplement: Supplementary file 1 [file marinedrugs-23-00167-s001.zip › marinedrugs-3567430-supplementary.pdf]

[Supplementary Figures]

Extracts from *Allium pseudojaponicum Makino* Target STAT3 Signaling Pathway to Overcome Cisplatin Resistance in Lung Cancer

Soo-Bin Nam [1,2,†], Jung Hoon Choi [3,4,†], Ga-Eun Lee [1], Jin Young Kim [3], Mee-Hyun Lee [5], Gabsik Yang [6], Yong-Yeon Cho [2], Hye Gwang Jeong [4], Geul Bang [3,*] and Cheol-Jung Lee [1,7,*]

[1] Biopharmaceutical Research Center, Korea Basic Science Institute (KBSI), Cheongju 28119, Republic of Korea; nsb0607@kbsi.re.kr (S.-B.N.); gelee3131@kbsi.re.kr (G.-E.L.)

[2] College of Pharmacy, The Catholic University of Korea, Bucheon 14662, Republic of Korea; yongyeon@catholic.ac.kr

[3] Digital Omics Research Center, Korea Basic Science Institute (KBSI), Cheongju 28119, Republic of Korea; jhchoi19@kbsi.re.kr (J.H.C.); jinyoung@kbsi.re.kr (J.Y.K.)

[4] College of Pharmacy, Chungnam National University, Daejeon 34134, Republic of Korea; hgjeong@cnu.ac.kr

[5] College of Korean Medicine, Dongshin University, Naju 58245, Republic of Korea; mhlee@dsu.ac.kr

[6] Department of Korean Medicine, College of Korean Medicine, Woosuk University, Jeonju 55338, Republic of Korea; yanggs@woosuk.ac.kr

[7] Department of Bio-Analytical Science, University of Science and Technology (UST), Dae-jeon 34113, Republic of Korea

*Correspondence: bangree@kbsi.re.kr (G.B.); veritas0613@kbsi.re.kr (C.-J.L.)

†These authors contributed equally to this work.

Supplementary Fig. 1

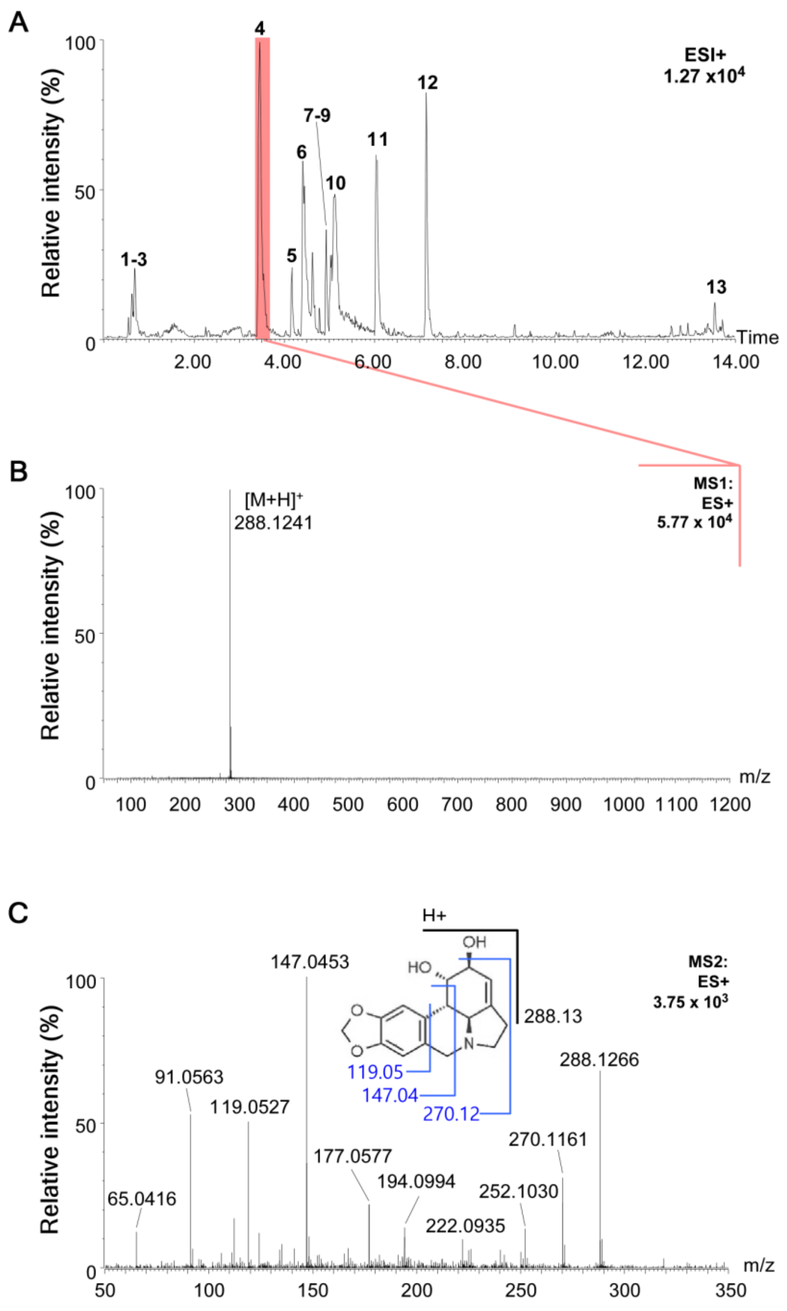

Fig. S1 Metabolic profiling of APE by UPLC/MS^E in positive ion mode. (A) Base peak ion chromatograms of the 50 μg APE. (B) The individual full-scan MS1 spectra of APE (red boxes). (C) Acquired MS/MS spectra of lycorine, the most abundant compound in APE, with the fragmentation pattern.

Supplementary Fig. 2

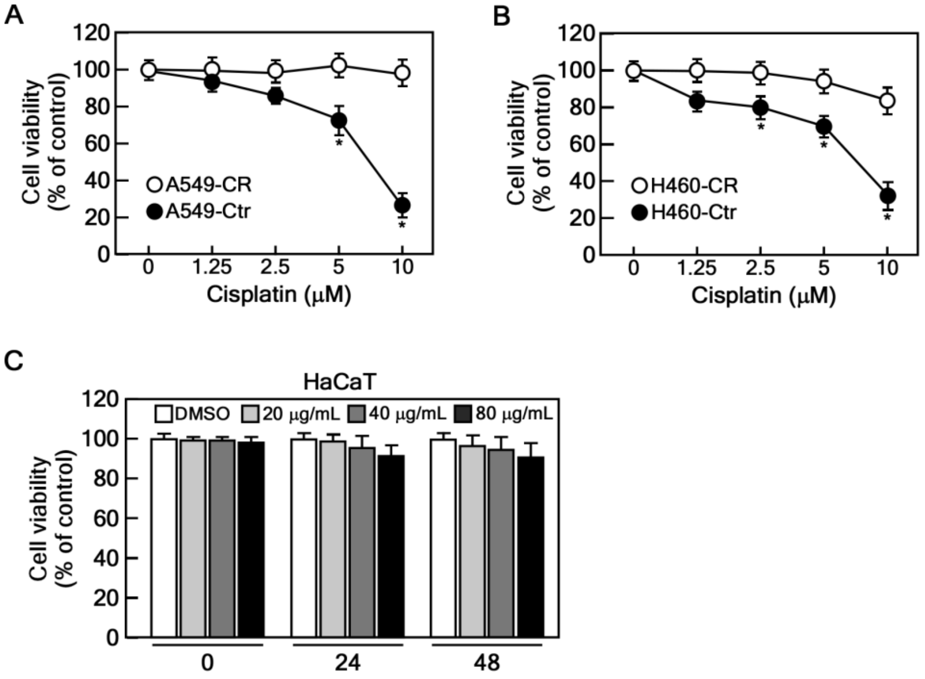

Fig. S2 Confirmation of cisplatin resistance in NSCLC cells and cytotoxicity of APE in HaCaT cells. (A, B) A549-Ctr, A549-CR, H460-Ctr and H460-CR cells (3 x 10^3cells/well) were seeded into 96-well plates and treated with 0, 1.25, 2.5, 5, 10 μM of cisplatin for 24h. (C) HaCaT cells (3 x 10^3cells/well) were seeded into 96-well plates and treated with 20, 40, 80 ug/ml of APE for 24 and 48 h. Cell viability was assessed using a CCK-8 assay.
